# Supplementary material for: Native flagellar MS ring is formed by 34 subunits with 23-fold and 11-fold subsymmetries
Source: Nat Commun. 2021 Jul 9;12:4223. doi: 10.1038/s41467-021-24507-9 (PMC8270960; doi:10.1038/s41467-021-24507-9)
Supplement: Supplementary file 1 — Supplementary Information [file 41467_2021_24507_MOESM1_ESM.pdf]

Supplementary Information

Native flagellar MS ring is formed by 34 subunits with 23-fold and 11-fold subsymmetries

Akihiro Kawamoto, Tomoko Miyata, Fumiaki Makino, Miki Kinoshita, Tohru Minamino, Katsumi Imada, Takayuki Kato and Keiichi Namba

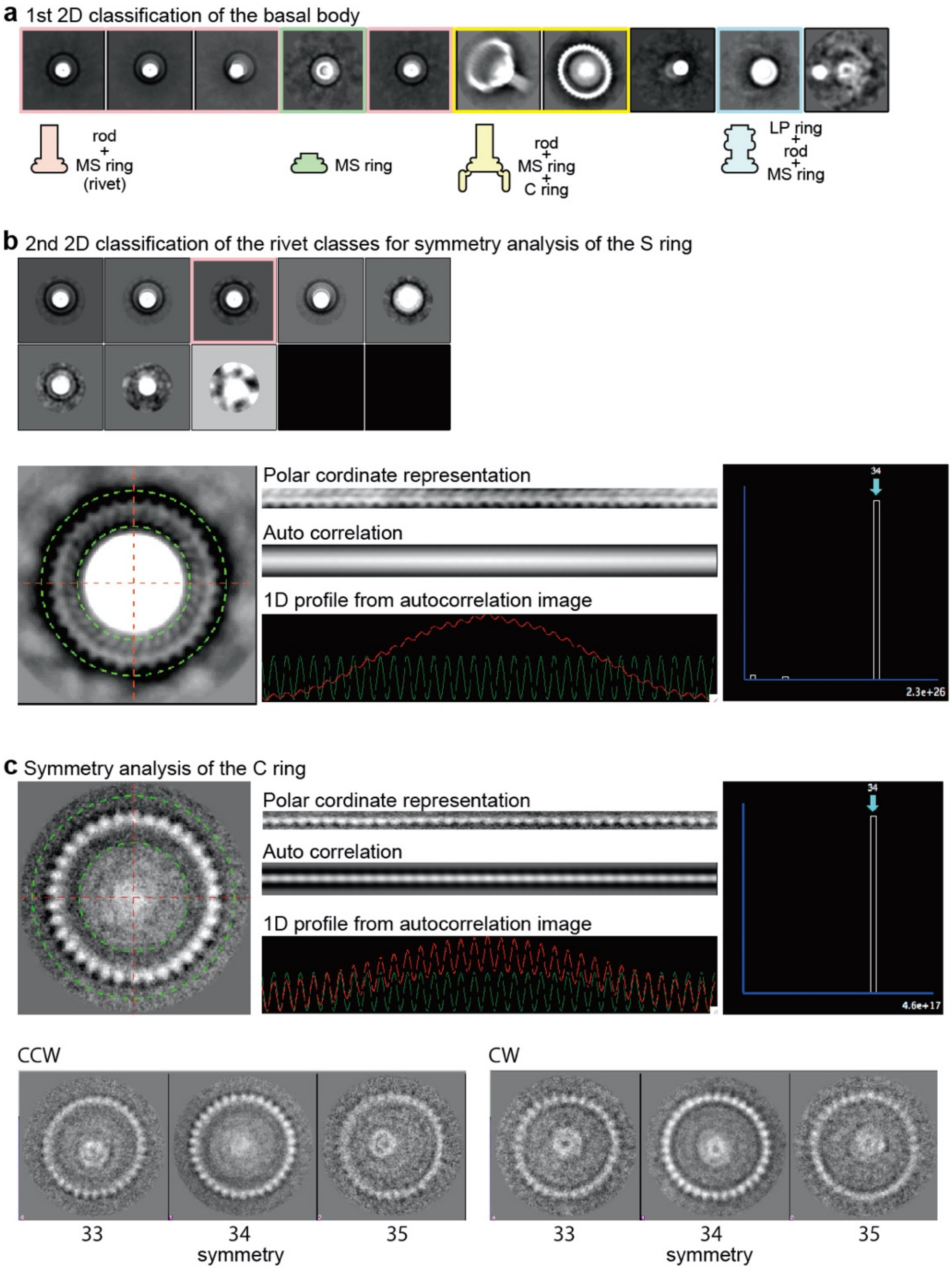

Supplementary Fig. 1 CryoEM single particle 2D Image analyses of the basal body M ring and C ring. a, 2D class average images of the basal body in the first round with identification of the types of substructures schematically depicted below. **b**, The upper panel shows the second round 2D class average for the images of the rivet class (pink boxes in **a**). The rotational symmetry of the S ring was analyzed by image analysis described in the lower panels. **c**, Symmetry analysis of the C ring with end-on view images. The upper panel describes how the image analysis is done, and the lower panels show C ring images of different rotational symmetries. WT: the wild-type motor in the CCW state; and CW: the CW-locked motor by FliG ΔPAA mutation[1]. The image presented here are averages of many particles for easy recognition of their symmetries, but the actual symmetry analysis was done with individual particle images used in a previous study[2].

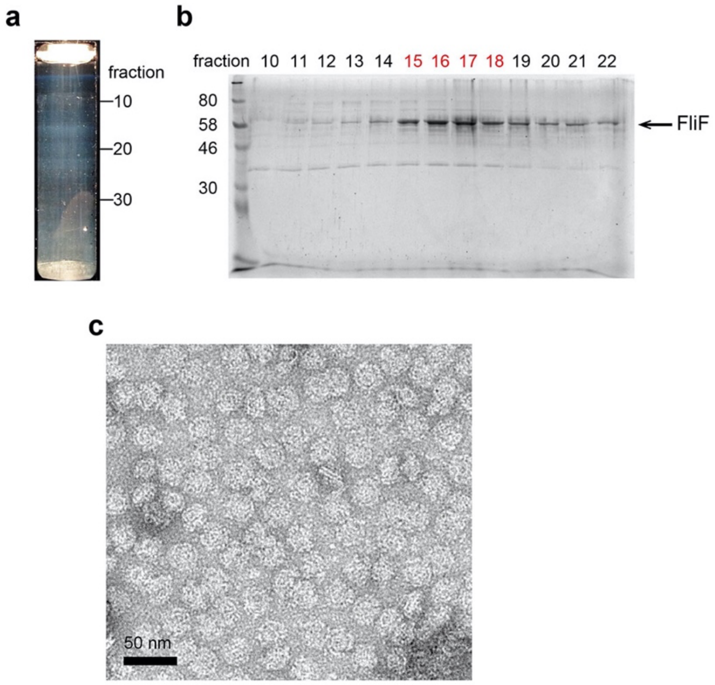

Supplementary Fig. 2 Purification of the MS ring formed by full-length FliF. a, The sucrose gradient for purification of the MS ring with fraction numbers on the right. **b**, SDS-PAGE band pattern of each fraction of the sucrose gradient shown in **a.** The positions of molecular mass markers (kDa) are shown on the left. **c**, Negative-stain EM image of the purified MS rings.

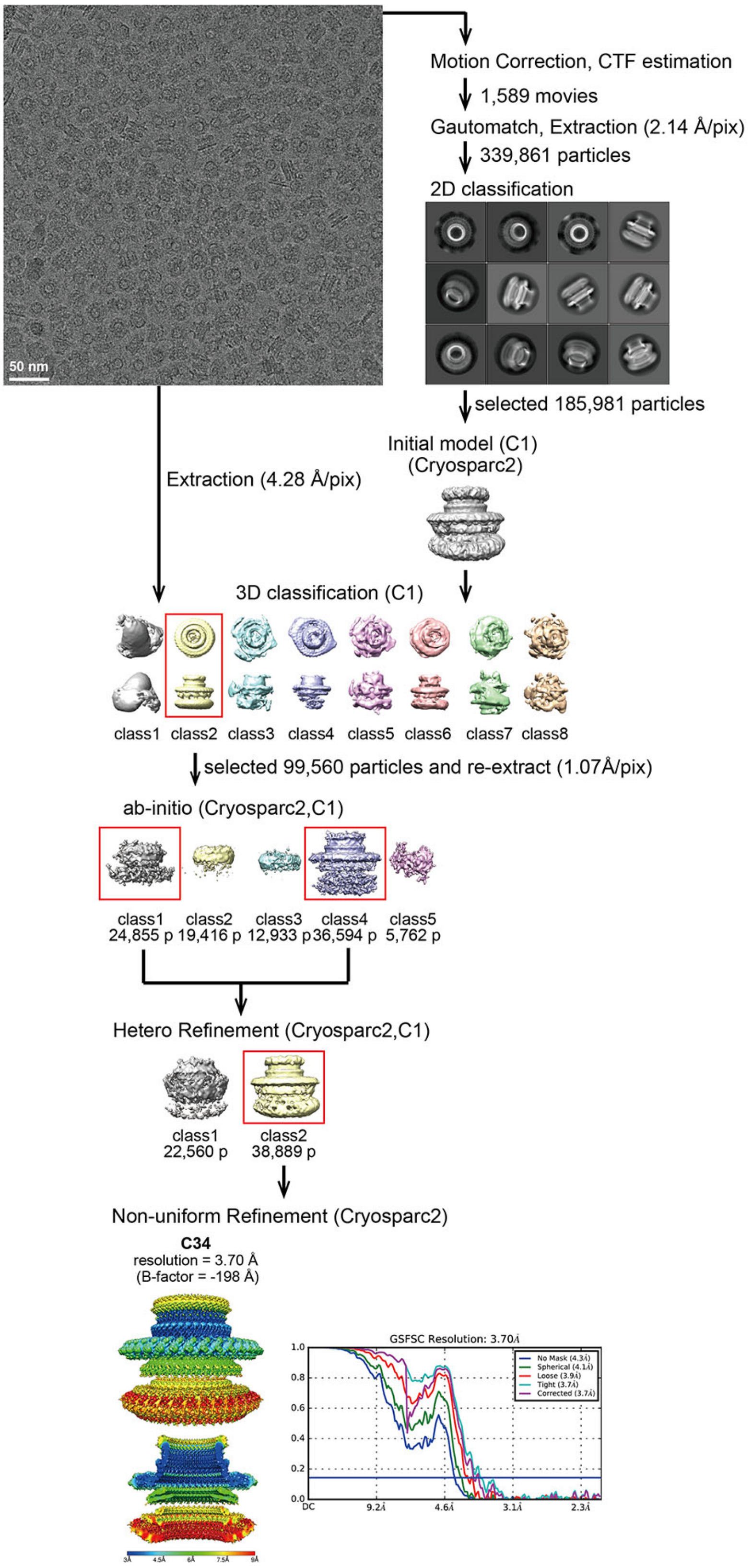

Supplementary Fig. 3 CryoEM single particle 3D image analysis of the MS ring formed by FliF expressed from a plasmid pKOT112 (ref. [3]) (data set 1).

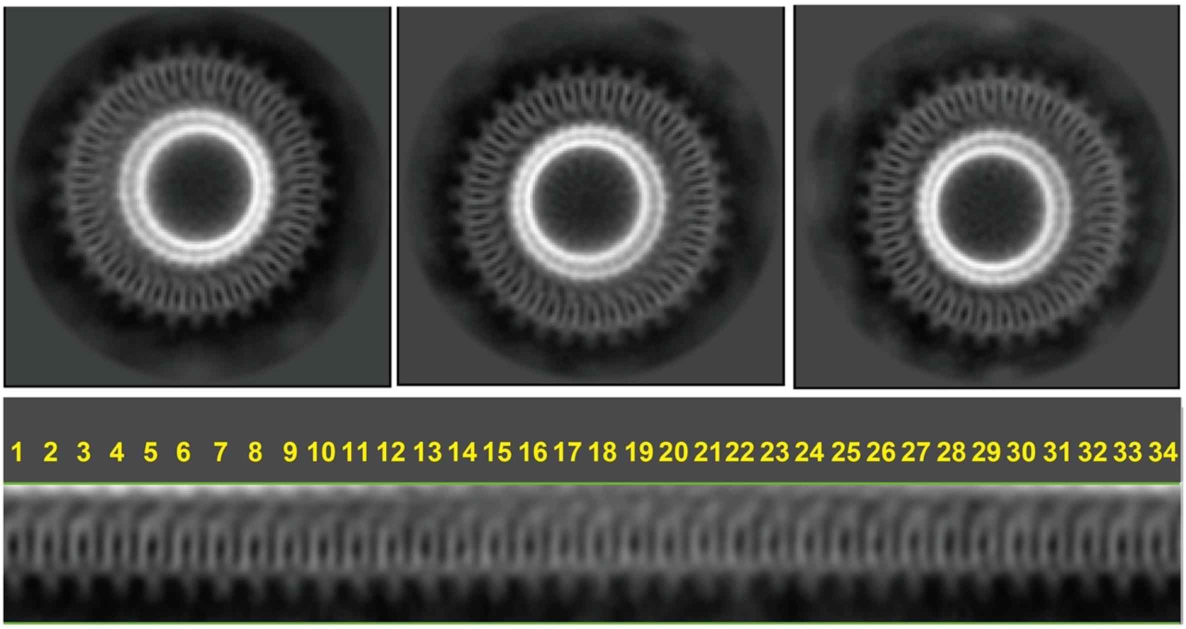

Supplementary Fig. 4 CryoEM single particle 2D image analyses of the MS ring. Upper three panels show 2D class average images of the MS ring in end-on views with slightly different orientation, which all clearly indicate 34-fold rotational symmetry of the S ring and collar, as analyzed by converting the images into the polar coordinates as shown in the lower panel.

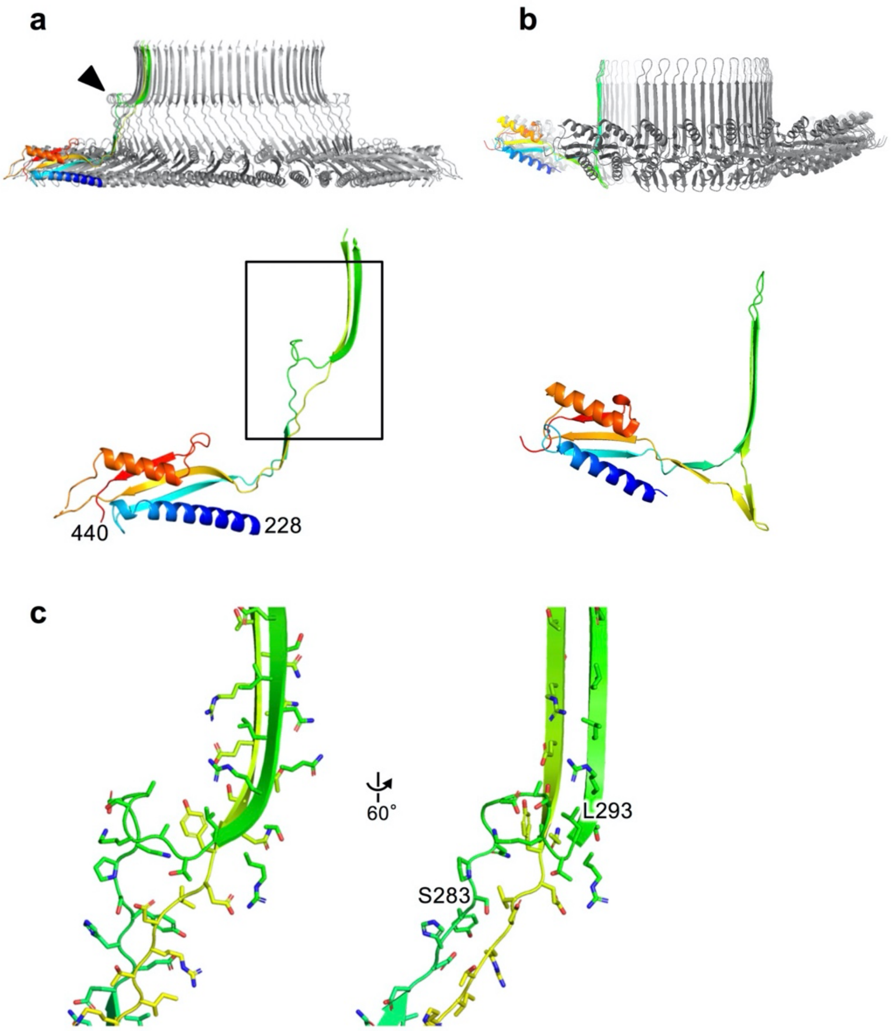

Supplementary Fig. 5 Structural comparison of the S ring and SpoIIIAG. **a**, the S ring; **b**, SpoIIIAG (PDB ID: 5WC3) in Cα ribbon representation with rainbow color according to the sequence from the N-terminus in blue to the C-terminus in red**. c**, Magnified views of part of FliF forming the collar above the S ring, as indicated by the square box in **a.**

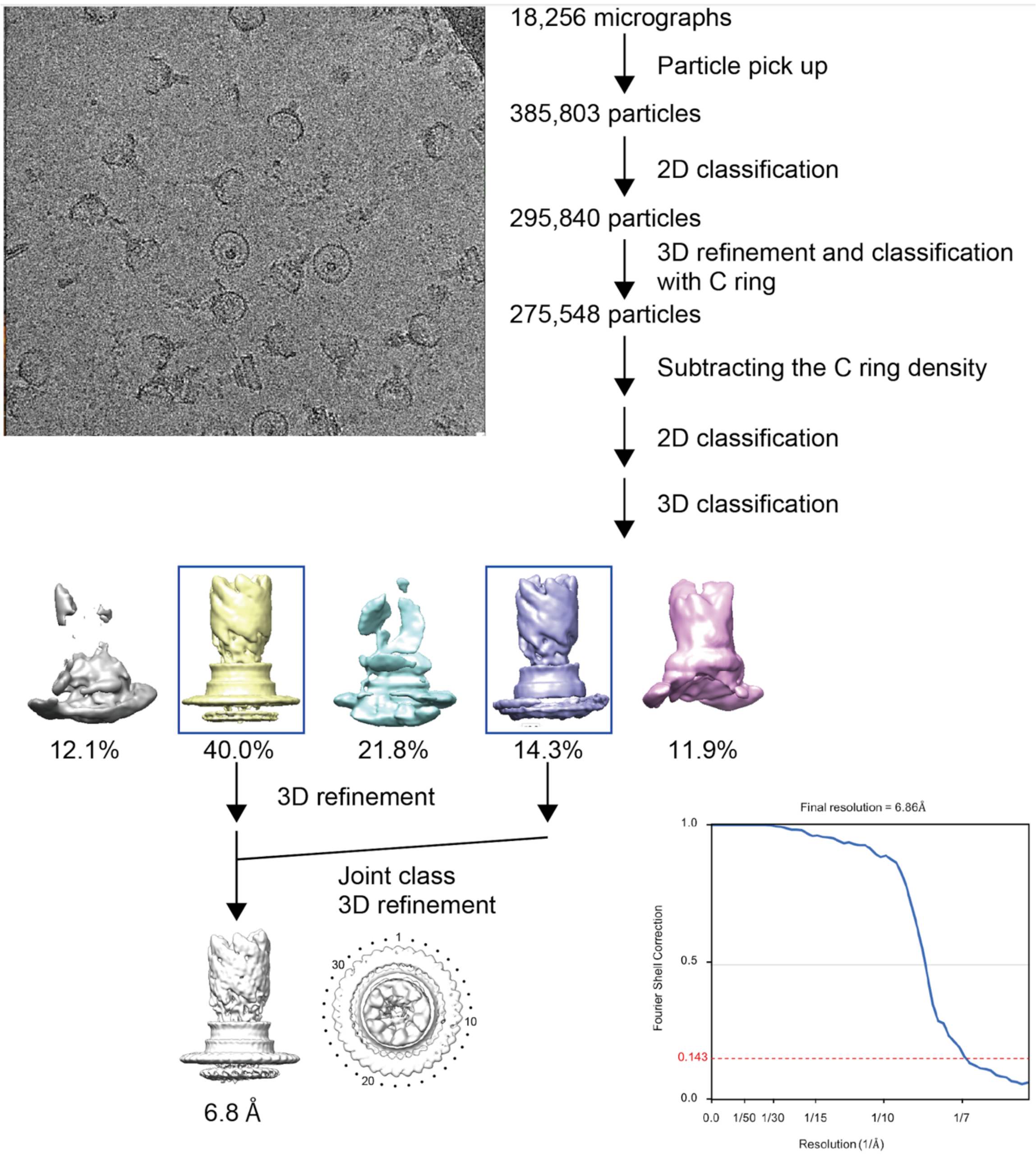

Supplementary Fig. 6 CryoEM single particle 3D image analysis of the flagellar basal body focusing on the MS ring and rod.

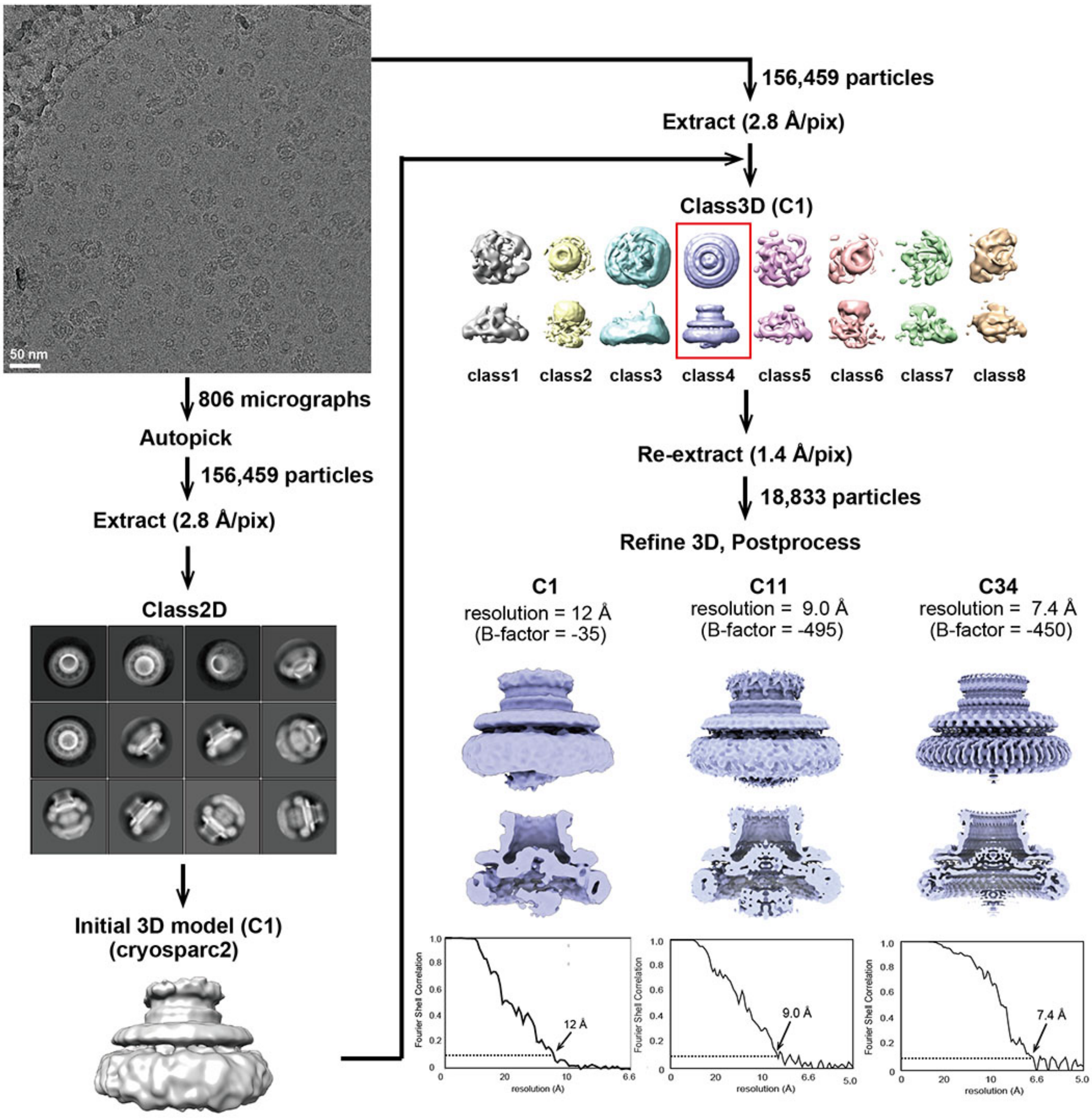

Supplementary Fig. 7 CryoEM single particle 3D image analysis of the MS ring formed by FliF expressed from a plasmid pKOT105 (ref.[4]) (data set 2).

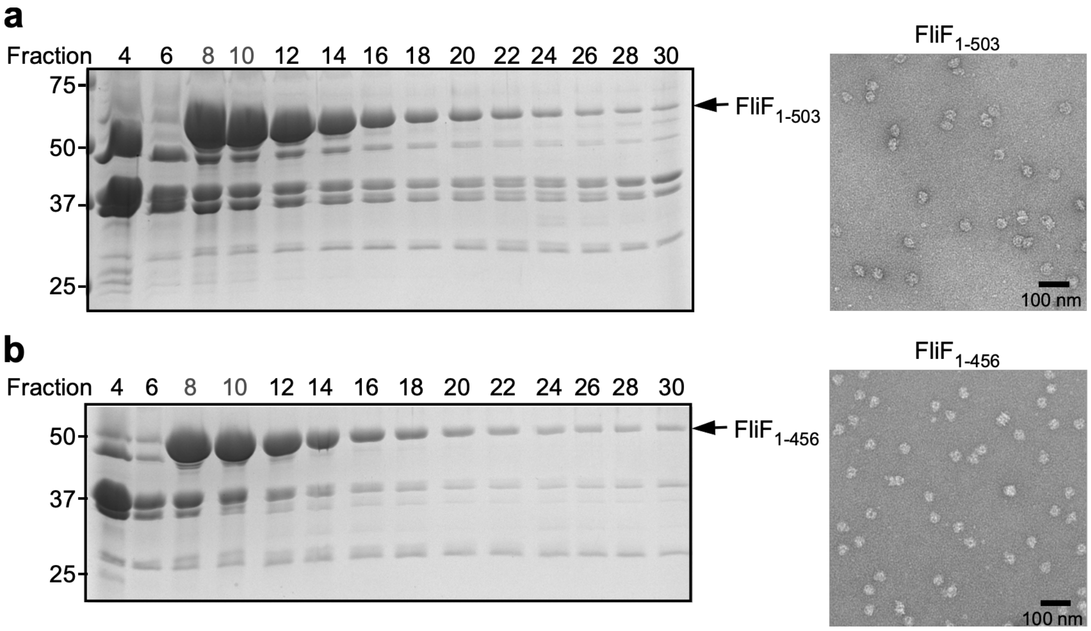

Supplementary Fig. 8 Purification of the MS ring formed by C-terminally-truncated fragments of FliF, FliF$_{1\text{-}503}$ and FliF$_{1\text{-}456}$. CBB staining SDS-PAGE gels of fractions containing (a) FliF$_{1\text{-}503}$ or (b) FliF$_{1\text{-}456}$ after sucrose gradient ultracentrifugation. The potions of molecular mass markers (kDa) are indicated on the left. Fractions 8, 9 and 10 were collected and concentrated by ultracentrifugation. The rings formed by FliF$_{1\text{-}503}$ or FliF$_{1\text{-}456}$ were observed by electron microscopy with negative staining.

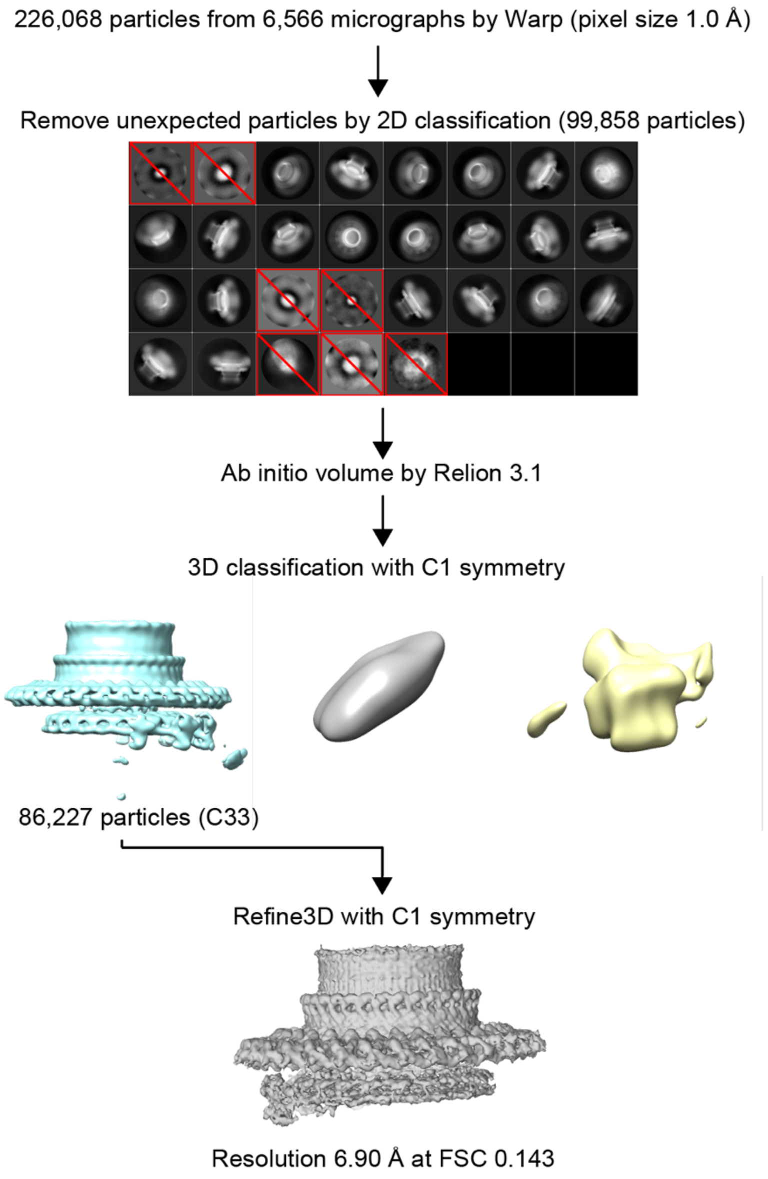

Supplementary Fig. 9 CryoEM single particle 3D image analysis of the MS ring formed by FliF$_{1\text{-}503}$.

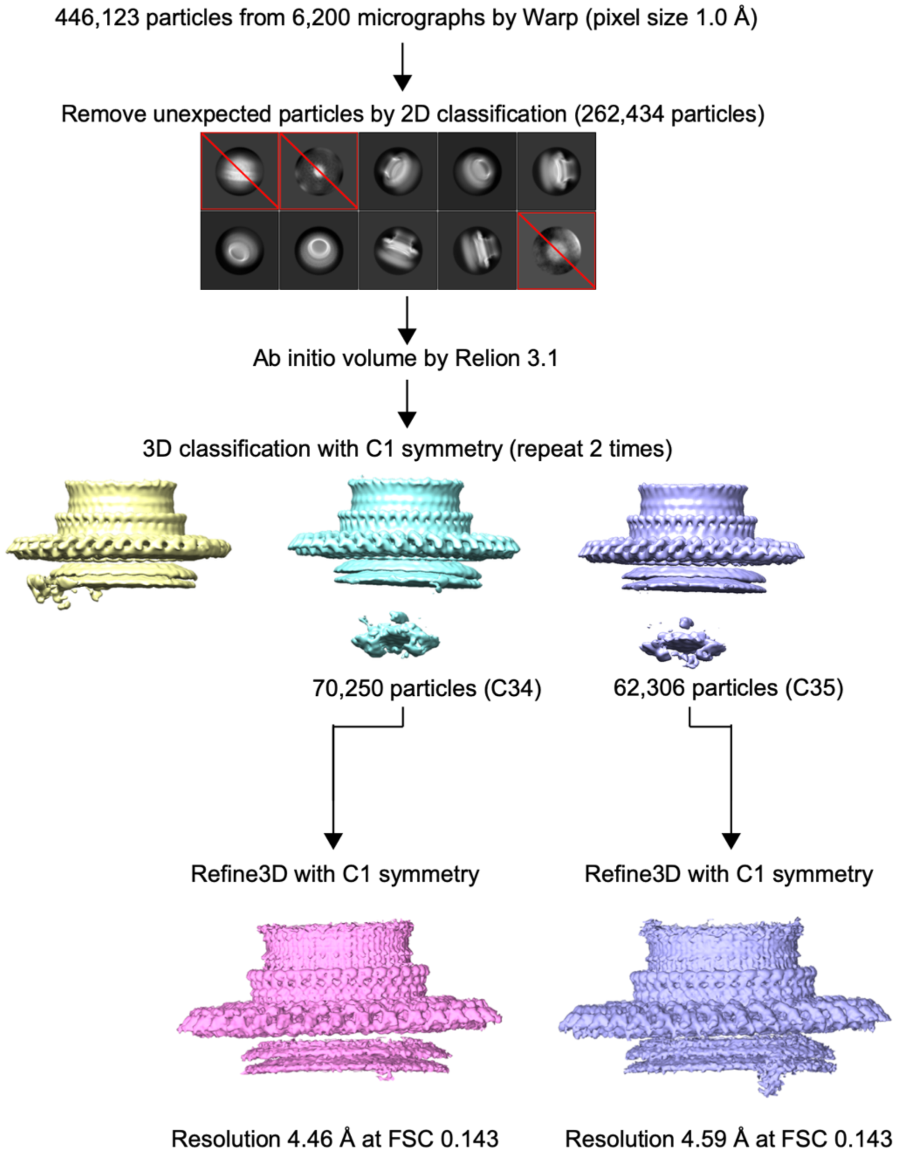

Supplementary Fig. 10 CryoEM single particle 3D image analysis of the MS ring formed by FliF$_{1\text{-}456}$.

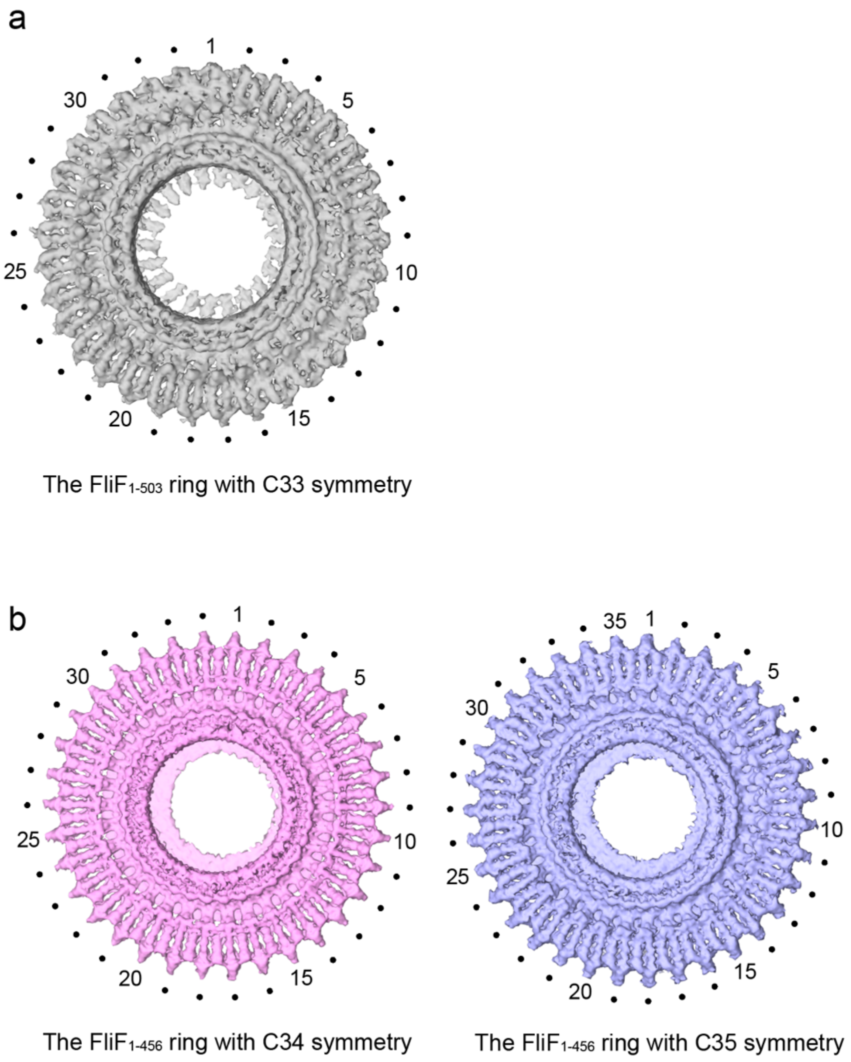

Supplementary Fig. 11 Rotational symmetries of the MS rings formed by FliF$_{1\text{-}503}$ and FliF$_{1\text{-}456}$ visualized in end-on views of their cryoEM 3D maps. a, The FliF$_{1\text{-}503}$ ring with C33 symmetry. **b,** The FliF$_{1\text{-}456}$ rings showing C34 and C35 symmetries.

Supplementary Table 1. Strains and plasmids used in this study

Strains and Plasmids	Relevant characteristics	Source or reference
Escherichia coli		
BL21 Star (DE3)	Over-expression of wild-type FliF and its mutant variants	Novagen
Salmonella		
SJW1368	Δ(*cheW-flhD*); master operon mutant	Ref. 2
TH12415	Δ*fliF7355*	Kelly. T. Hughes
HK1003	*flgE*Δ*(9-20)* Δ*clpP*::Cm	Hideyuki Matsunami
TM022	*flgE*Δ*(9-20)* Δ*clpP*::Cm *fliG*ΔPAA	Ref. 3
Plasmids		
pKOT105	pET3b / FliF	Ref. 4
pKOT112*	pET3b / FliF	Ref. 5
pET3c	Expression vector	GE Healthcare
pMKMiF001	pET3c / FliF	This study
pMKMiF001TH	pET3c / FliF-TEV-His_{10}	This study
pMKMiF002	pET3c / FliF(I252A)	This study
pMKMiF00TH	pET3c / FliF(I252A)-TEV-His_{10}	This study
pMKMiF003	pET3c / FliF(I252R)	This study
pMKMiF003TH	pET3c / FliF(I252R)-TEV-His_{10}	This study
pMKMiF004	pET3c / FliF(L253A)	This study
pMKMiF004TH	pET3c / FliF(L253A)-TEV-His_{10}	This study
pMKMiF005	pET3c / FliF(L253R)	This study
pMKMiF005TH	pET3c / FliF(L253R)-TEV-His_{10}	This study
pMKMiF006	pET3c / FliF(V266A)	This study
pMKMiF006TH	pET3c / FliF(V266A)-TEV-His_{10}	This study
pMKMiF007	pET3c / FliF(V266R)	This study
pMKMiF007TH	pET3c / FliF(V266R)-TEV-His_{10}	This study
pMKMiF008	pET3c / FliF(I252C)	This study
pMKMiF009	pET3c / FliF(H263C)	This study
pMKMiF0010	pET3c / FliF(A388C)	This study
pMKMiF011	pET3c / FliF(I252C/H263C)	This study
pMKMiF011SP-1	pET3c / FliF(I252C/H263Y)	This study
pMKMiF012	pET3c / FliF(I252C/A388C)	This study
pMKMiF013	pTrc99AFF4/$FliF_{(1-503)}$	This study
pMKMiF014	pTrc99AFF4/$FliF_{(1-456)}$	This study

***pKOT112 was supposed to contain a sequence corresponding to a C-terminally-truncated FliF fragment (Ser-1 to Asp-456) but the plasmid we received actually contained full-length FliF.**

Supplementary Table 2 CryoEM data, model refinement and validation statistics

**Data collection and processing**	FliF ring (data set 1)<br>(EMDB-30612)<br>(PDB 7D84)	FliF ring (data set 2)<br>(EMDB-30363, EMDB-30361, EMDB-30360)			basal body<br>(EMDB-30613)
Magnification	75,000	59,000			59,000
Voltage (kV)	300	300			300
Electron exposure<br>(e^{-} $Å^{-2}$)	90	90			90
Defocus range (μm)	1.0-3.0	1.0-3.0			0.5-3.5
Pixel size (Å)	1.07	1.4			1.4
Symmetry imposed	C34	C1	C11	C34	C1
Initial particle images (no.)	339,861	156,459			385,803
Final particle images (no.)	38,889	18,833			149,341
Map resolution (Å)<br>FSC threshold	3,70<br>0.143	12.0<br>0.143	9.0<br>0.143	7.4<br>0.143	6.8<br>0.143
Map resolution range (Å)	3.0-9.0				
**Refinement**					
Initial model used (PDB code)	-				
Model resolution (Å)<br>FSC threshold	4.2<br>0.5				
Model resolution range (Å)	3.7-4.2				
Map sharpening *B* factor ($Å^2$)					
Model composition<br>Non-hydrogen atoms<br>Protein residues<br>Ligands	 42,704<br>42,704<br>0				
B factors ($Å^2$)<br>Protein<br>Ligand	 90.68<br>-				
r.m.s. deviations<br>Bond lengths (Å)<br>Bond angles (°)	 0.009<br>0.882				
Validation<br>MolProbity score<br>Clashscore<br>Poor rotamers (%)	 1.96<br>4.82<br>7.78				
Ramachandran plot<br>Favored (%)<br>Allowed (%)<br>Disallowed (%)	 97.87<br>7.78<br>0				

Supplementary Table 3 CryoEM data and analysis

**Data collection and processing**	$FliF_{1\text{-}503}$ ring (EMDB-30940)	$FliF_{1\text{-}456}$ ring (EMDB-30941, EMDB-30942)	
Magnification	50,000	50,000	50,000
Voltage (kV)	300	300	300
Electron exposure (e^{-} $Å^{-2}$)	50	50	50
Defocus range (μm)	0.5-2.5	0.5-2.5	0.5-2.5
Pixel size (Å)	2.0	2.0	2.0
Symmetry imposed	C1	C1	C1
Initial particle images (no.)	226,068	446,123	446,123
Final particle images (no.)	86,227	62,306	70,250
Map resolution (Å)<br>FSC threshold	6.9<br>0.143	4.59<br>0.143	4.36<br>0.143

Supplementary Table4. Primer sequences used in this study

Primer name	Sequence (5' to 3')
NdeI_FliF_F	GGGAATTCCATATGAGTGCGACTGCATCGACT
FliF_BamHI_R	CGCGGATCCTTACTCATGATCGTTACTCATC
FliF_TEV_His$_{10}$_BamHI_R	CGCGGATCCTTAGTGATGATGATGATGATGATGATGATGATGGCCCTGAAAATACAGGTTTTCCTCATGATCGTTACTCATCC
FliF_I252A_F	CGCCGTATCGAAGCCGCTCTGTCGCCTATCGTC
FliF_I252A_R	GACGATAGGCGACAGAGCGGCTTCGATACGGCG
FliF_I252R_F	CGCCGTATCGAAGCCCGTCTGTCGCCTATCGTC
FliF_I252R_R	GACGATAGGCGACAGACGGGCTTCGATACGGCG
FliF_L253A_F	CGTATCGAAGCCATTGCGTCGCCTATCGTCGGG
FliF_L253A_R	CCCGACGATAGGCGACGCAATGGCTTCGATACG
FliF_L253R_F	CGTATCGAAGCCATTCGGTCGCCTATCGTCGGG
FliF_L253R_R	CCCGACGATAGGCGACCGAATGGCTTCGATACG
FliF_V266A_F	AATGTTCACGCTCAGGCAACCGCCCAGTTGGAT
FliF_V266A_R	ATCCAACTGGGCGGTTGCCTGAGCGTGAACATT
FliF_V266R_F	AATGTTCACGCTCAGCGAACCGCCCAGTTGGAT
FliF_V266R_R	ATCCAACTGGGCGGTTCGCTGAGCGTGAACATT
FliF_I252C_F	CGCCGTATCGAAGCCTGTCTGTCGCCTATCGTC
FliF_I252C_R	GACGATAGGCGACAGACAGGCTTCGATACGGCG
FliF_H263C_F	GGGAACGGTAATGTTTGCGCTCAGGTAACCGCC
FliF_H263C_R	GGCGGTTACCTGAGCGCAAACATTACCGTTCCC
FliF_A388C_F	GAGCGTCTCTCCGTGTGCGTCGTGGTGAATTAC
FliF_A388C_R	GTAATTCACCACGACGCACACGGAGAGACGCTC
FliF503_BamHI_R	CGCGGATCCTTAATCGATAAAGGACTG
FliF456_BamHI_R	CGCGGATCCTTAATCGATAAAGGACTG

***F and R mean forward and reverse primers.**

References

1. Togashi, F., Yamaguchi, S., Kihara, M., Aizawa, S.I. & Macnab, R.M. An extreme clockwise switch bias mutation in *fliG* of *Salmonella typhimurium* and its suppression by slow-motile mutations in motA and motB. *J Bacteriol* **179**, 2994-3003 (1997).
2. Ohnishi, K., Ohto, Y., Aizawa, S.-I., Macnab, R.M. & Iino, T. FlgD is a scaffolding protein needed for flagellar hook assembly in *Salmonella typhimurium. J. Bacteriol.* **176**, 2272–2281 (1994).
3. Sakai, T. *et al*. Novel Insights into Conformational Rearrangements of the Bacterial Flagellar Switch Complex. *mBio* **10**, e00079-19 (2019).
4. Ueno, T., Oosawa, K. & Aizawa, S. M ring, S ring and proximal rod of the flagellar basal body of *Salmonella typhimurium* are composed of subunits of a single protein, FliF. *J Mol Biol* **227**, 672-677 (1992).
5. Ueno, T., Oosawa, K. & Aizawa, S. Domain structures of the MS ring component protein (FliF) of the flagellar basal body of *Salmonella typhimurium*. *J Mol Biol* **236**, 546-555 (1994).
